# Multifocal Colonic Masses as a Presentation of Secondary Extramedullary Plasmacytoma in Relapsing‐Refractory Multiple Myeloma: A Case Report and Review of Literature

**DOI:** 10.1002/jgh3.70239

**Published:** 2025-08-29

**Authors:** Sirisha Gaddipati, Sunny Sandhu, Eric F. Martin

**Affiliations:** ^1^ Department of Medicine University of Miami Miller School of Medicine Miami Florida USA; ^2^ Division of Digestive Health and Liver Diseases, Department of Medicine Miller School of Medicine at the University of Miami Miami Florida USA

**Keywords:** colon metastasis, extramedullary plasmacytoma, multiple myeloma, plasmacytoma

## Abstract

**Background:**

Multiple myeloma (MM) is a common plasma cell malignancy; however, extramedullary multiple myeloma (EMM) is an aggressive phenotype in which malignant plasma cells proliferate outside of the bone marrow.

**Aims:**

Extramedullary plasmacytomas (EMP) are uncommon and gastrointestinal involvement is exceedingly rare.

**Case Presentation:**

Here, we present a case of multifocal secondary colonic EMP in a patient with relapsed refractory multiple myeloma (RRMM) and review literature demonstrating other cases of secondary colonic EMP.

## 1 Introduction

Multiple myeloma (MM) is a common plasma cell malignancy, accounting for approximately 15% of all hematologic malignancies [1]. While MM characteristically involves the bone marrow, malignant plasma cells can also proliferate outside of the bone marrow, which is a rare and aggressive phenotype termed extramedullary multiple myeloma (EMM) [2]. When extramedullary malignant plasma cells present as a solitary tumor, they are termed plasmacytoma. Extramedullary plasmacytomas (EMP) account for only 4% of all plasma cell neoplasms [3]. Gastrointestinal involvement, especially colon involvement, is exceedingly rare. Secondary colonic EMPs, which are plasma cell tumors arising in cases of systemic MM, have only been reported in 11 other cases [4, 5]. Here, we present a case of multifocal secondary colonic EMP in a patient with relapsed refractory multiple myeloma (RRMM) and perform a review of other cases described in the literature.

## 2 Case Presentation

**FIGURE 1:**
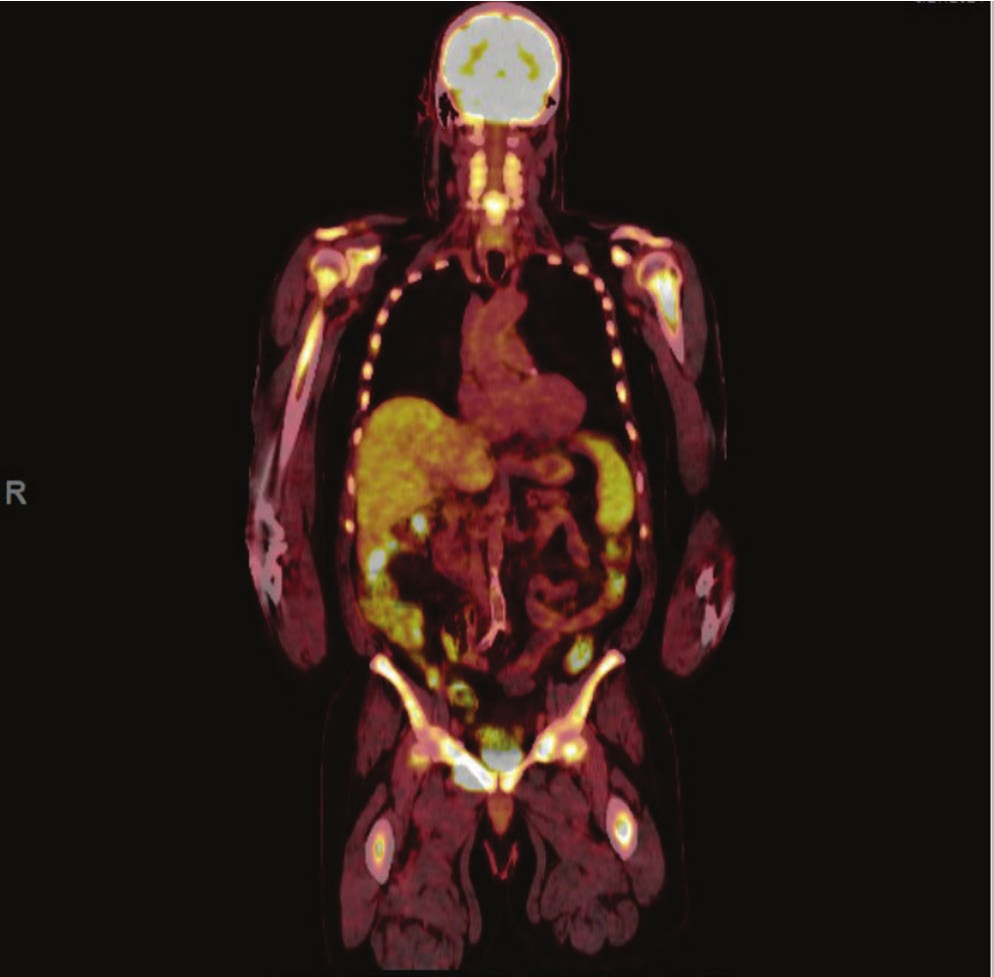
Coronal section of PET scan with focal uptake of pleural‐based nodules, diffuse increased metabolic activity throughout the splenic parenchyma, and focal abnormal metabolic activity is noted within the hepatic flexure and sigmoid colon.

**FIGURE 2:**
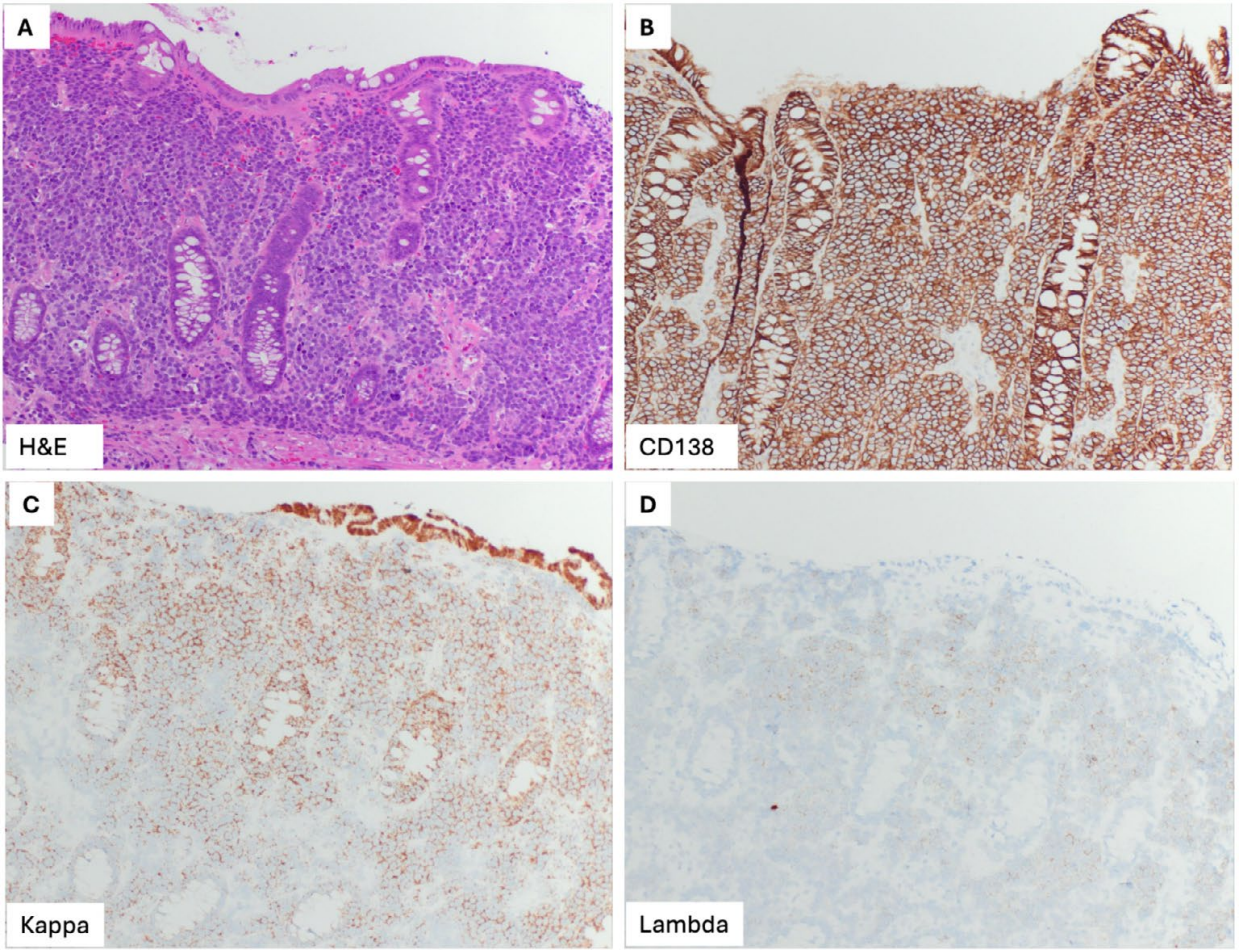
Histologic findings of ascending colon mass. The large intestinal mucosa is involved by sheets of plasma cells (A). The plasma cells are positive for CD138 (B), and are kappa‐restricted by kappa and lambda in situ hybridization stain (C and D).

**FIGURE 3:**
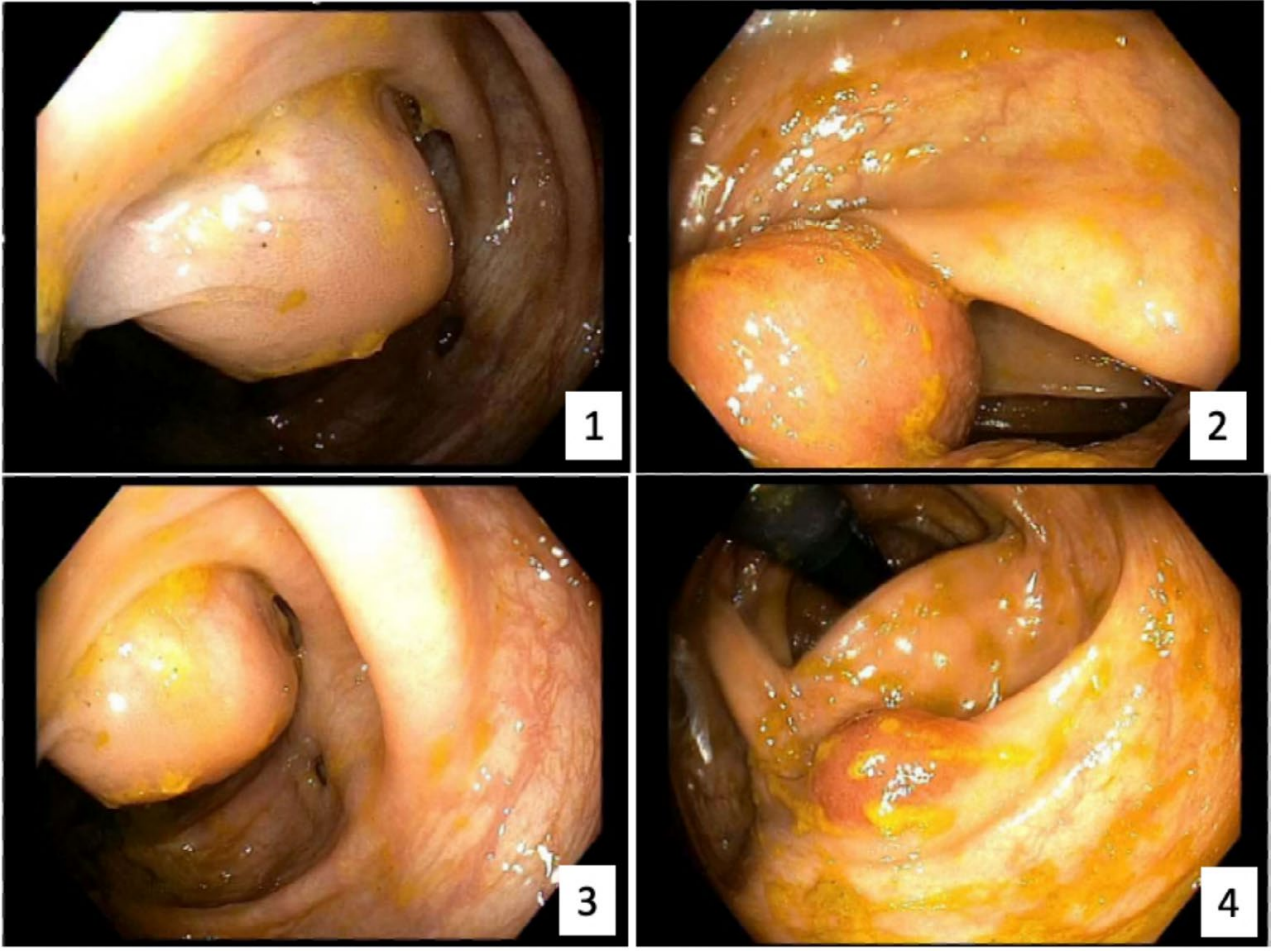
Evidence of polyps on initial colonoscopy including ascending colon (1–3) and cecum (4).

A 70‐year‐old man with diabetes mellitus type 2 and hypertension had laboratory studies which showed paraproteinemia with IgG‐kappa level of 4040 g/dL and thrombocytopenia with platelets 101 000/uL. Bone marrow biopsy demonstrated 60% plasma cells, confirming the diagnosis of multiple myeloma (MM). Positron emission tomography (PET) scan demonstrated diffuse activity in the axial and proximal appendicular skeleton. He was started on chemotherapy with daratumumab and carfilzomib, lenalidomide, and dexamethasone for 9 months. Subsequent laboratory testing demonstrated persistent elevation of serum free light chains, and repeat bone marrow biopsy demonstrated 90% plasma cells. He was enrolled in a clinical trial of chemotherapy; however, he developed progression of disease, with repeat PET scan showing diffuse uptake in the bone marrow with superimposed focal lesions, pleural‐based nodules, pulmonary masses, and increased focal metabolic activity within the hepatic flexure and sigmoid colon (Figure 1). He reported new onset constipation but denied any other gastrointestinal complaints. Diagnostic colonoscopy revealed a large polypoid, partially circumferential, non‐obstructive mass in the distal ascending colon involving two‐thirds of luminal circumference with a luminal diameter of 10 mm, with several biopsies performed. In addition, there was also a 17 mm sessile polyp in the cecum and a 4 mm semi‐pedunculated polyp in the transverse colon, and both were also biopsied (Figure 2). Pathology from all the lesions demonstrated similar morphologic features, showing intestinal mucosa involved by sheets of plasma cells which were positive for CD138 and MUM1, and were kappa restricted by kappa and lambda in situ hybridization, consistent with plasmacytoma (Figure 3). He was ultimately diagnosed with plasma cell leukemia and began treatment with etoposide, prednisone, vincristine, cyclophosphamide, and doxorubicin (EPOCH) as bridging therapy prior to CAR‐T cell infusion for treatment of RRMM and currently remains on CAR‐T therapy.

## 3 Discussion

Plasma cell neoplasms can present in a variety of distinct manifestations including typical MM, lymphoplasmacytic lymphoma, solitary plasmacytoma of the bone, and extramedullary plasmacytomas [6]. While primary EMPs are more common and arise in the absence of systemic MM, secondary EMPs are considered a rare extramedullary manifestation of MM and have mostly been described in isolated case reports. In cases of relapsing MM, the incidence can be as high as 10%, although data is lacking given the overall limited cases [1]. While osseous sites (namely vertebrae, ribs, sternum, skull, and pelvis) are most commonly described in secondary EMP, other less common sites include liver, kidney, lymph nodes, central nervous system, breast, pleura, and pericardium [7, 8]. Gastrointestinal involvement, however, is extremely rare in secondary EMP [5]. Presentation can be variable, with reported cases describing intussusception, constipation, bowel obstruction, and rectal bleeding. Diagnosis of colonic involvement can be established by endoscopic evaluation with biopsies to rule out alternative and more common colonic malignancies. Due to its infrequent occurrence, there is no current standard treatment for colonic EMP. Given the systemic involvement of the underlying malignancy, chemotherapy is typically the mainstay of treatment, and surgery is typically avoided other than in cases of colonic obstruction. While the overall prognosis of intestinal EMP is unclear due to its relative rarity, EMP involvement is associated with poorer outcomes—in a retrospective study on transplant‐eligible patients with MM, 5‐year overall survival was 63% in patients with EMP and bone‐related plasmacytoma, compared to the 80% survival in those without plasmacytoma at MM diagnosis [8].

In our literature review of secondary EMP cases, we found 11 cases reported to date of isolated colonic involvement, with most cases reported as IgA predominant etiology (Table 1). The average age at presentation was 63.2 years, and most cases (63.6%) occurred in males. Colonic lesions were typically described as unifocal masses, with three cases involving the ascending colon, one case in the transverse colon, one in the descending colon, and five cases with rectal or sigmoid involvement. However, extensive and multifocal colonic involvement, as seen in our case, has only been reported in 1 other case, although the diagnosis was made on PET scan, and diagnostic colonoscopy was not pursued [9].

**TABLE 1:** Cases of secondary extramedullary plasmacytoma with colonic involvement.

Author (year)	Age	Sex	Type of MM	Treatment	Location of colonic mass	Symptoms
Kakati et al. (2012) [9]	57	M	IgA lambda LC	Chemotherapy Auto‐HSCT Salvage chemotherapy	Extensive: ascending, transverse, descending, sigmoid colon	None
Islam et al. (2010) [10]	52	M	Not specified	Chemotherapy Auto‐HSCT	Lower sigmoid	Hematochezia
Herbst et al. (2008) [11]	79	M	IgA	Colonic resection	Ascending colon mass	Anemia Constipation
Parnell (2015) [5]	72	F	IgM K	Prior txt: chemo R hemicolectomy, distal Ileal resection Chemo	Ulcerating mass in descending colon near hepatic flexure	Fatigue Dark stool
Fagkrezos (2018) [12]	71	F	Not specified	Prev: Chemotherapy L colectomy	Sigmoid mass (extension into fatty tissue, colonic perf)	Abdominal pain Pneumoperitoneium
Jones (2008) [13]	65	M	IgA lambda plasmacytoma	Sigmoid colon resection	Sigmoid mass	Dysuria, hematuria
Elias (1969) [14]	67	M	Diffuse IgG and IgA	L‐PAM, prednisone Radiotherapy	Transverse colon	Abdominal masses Rectal bleeding Large bowel obstruction
Elias (1969) [14]	58	M	MM (not specified)	Radiation therapy	Rectal mass	Large bowel obstruction
Mesa Lopez et al. (2022) [15]	71	F	IgG, Kappa LC	Chemotherapy	Ascending	IDA Positive FOBT
Venizelos (2004) [16]	61	F	IgG	Chemotherapy	Polypoid Rectal tumor	Lower back pain Fatigue
Triantafyllopoulou (2015) [17]	43	M	Not specified	Expired prior to treatment	Ascending colon	Colicky abdominal pain Anemia
Otrock (2006) [18]	50	M	Lambda LC	Expired prior to treatment	Diffuse colonic involvement with omental involvement	Abdominal pain and distension
Griffiths (1997) [19]	74	W	Lambda paraproteinemia	Hemicolectomy	Ascending colon	Abdominal pain Rectal bleeding Constipation

In summary, while colonic EMPs are rarely the etiology of multifocal masses on colonoscopy, clinicians should be aware of this differential in patients with a history of multiple myeloma [20]. Patients can often have subtle gastrointestinal symptoms which can be commonly overlooked. Extramedullary disease typically represents significant clinical progression of MM and portends a higher mortality rate; therefore, prompt recognition and treatment of colonic EMPs is crucial [21].

## Consent

Patient informed consent was obtained for case publication.

## Conflicts of Interest

The authors declare no conflicts of interest.
